# Supplementary material for: Mapping the evolution of fertility support policies in China: A content and instrumental analysis
Source: PLoS One. 2025 Oct 9;20(10):e0332137. doi: 10.1371/journal.pone.0332137 (PMC12510515; doi:10.1371/journal.pone.0332137)
Supplement: S1 Appendix — (ZIP) [file pone.0332137.s001.zip › S1 Appendix. 226 original policy documents/86-国务院未成年人保护工作领导小组关于印发《国务院未成年人保护工作领导小组关于加强未成年人保护工作的意见》的通知(FBM-CLI-4-5015092).doc]

国务院未成年人保护工作领导小组关于印发《国务院未成年人保护工作领导小组关于加强未成年人保护工作的意见》的通知

**发布部门：** [国务院未成年人保护工作领导小组](https://www.pkulaw.com/law/chl?Aggs.IssueDepartment=70605E3)

**发文字号：**国未保组〔2021〕1号

**发布日期：**2021.06.06

**实施日期：**2021.06.06

**时效性：** [现行有效](https://www.pkulaw.com/law/chl?Aggs.TimelinessDic=01)

**效力级别：** [部门规范性文件](https://www.pkulaw.com/law/chl?Aggs.EffectivenessDic=XE0302)

**法规类别：** [老少妇幼残保护](https://www.pkulaw.com/law/chl?Aggs.Category=01604) [营商环境优化](https://dobusiness.pkulaw.com/)

**专题分类：** [未成年人保护](https://www.pkulaw.com/law/chl?Aggs.SpecialType=015)

国务院未成年人保护工作领导小组关于印发《国务院未成年人保护工作领导小组关于加强未成年人保护工作的意见》的通知
（国未保组〔2021〕1号）

各省、自治区、直辖市未成年人保护工作领导小组（委员会），新疆生产建设兵团未成年人保护工作领导小组，国务院未成年人保护工作领导小组各成员单位：
　　《国务院未成年人保护工作领导小组关于加强未成年人保护工作的意见》已商中央编办同意，并经国务院未成年人保护工作领导小组第一次全体会议审议通过，现予印发，请认真贯彻落实。

国务院未成年人保护工作领导小组
　　2021年6月6日

国务院未成年人保护工作领导小组关于加强未成年人保护工作的意见

　　未成年人保护工作关系国家未来和民族振兴。党中央始终高度重视未成年人工作，关心未成年人成长。习近平总书记多次指出，少年儿童是祖国的未来，是中华民族的希望，强调培养好少年儿童是一项战略任务，事关长远。为深入学习贯彻习近平总书记重要指示批示精神，贯彻落实党中央、国务院关于加强未成年人保护工作决策部署，推动《[中华人民共和国未成年人保护法](https://www.pkulaw.com/chl/883cecd7fe38636dbdfb.html)》等法律法规落地落细，现就加强未成年人保护工作提出如下意见：
　　**一、**总体要求
　　（一）指导思想。
　　以习近平新时代中国特色社会主义思想为指导，深入学习贯彻习近平总书记关于未成年人保护工作重要指示批示精神，全面贯彻落实党的十九大、十九届二中、三中、四中、五中全会精神和党中央、国务院关于未成年人保护工作决策部署，立足新发展阶段、贯彻新发展理念、构建新发展格局，以满足人民日益增长的美好生活需要为根本目的，进一步加强组织领导、完善运行机制、强化制度建设、健全服务体系，切实保护未成年人身心健康、保障未成年人合法权益。
　　（二）基本原则。
　　--坚持党对未成年人保护工作的领导。把党的领导贯穿未成年人保护工作全过程各方面，紧紧围绕统筹推进“五位一体”总体布局和协调推进“四个全面”战略布局，坚持思想道德教育和权益维护保障相融合，大力培育和践行社会主义核心价值观，培养有理想、有道德、有文化、有纪律的社会主义建设者和接班人，培养担当民族复兴大任的时代新人。
　　--坚持最有利于未成年人的原则。以依法保障未成年人平等享有生存权、发展权、受保护权和参与权等权利，促进未成年人全面健康成长作为出发点和落脚点，在制定法律法规、政策规划和配置公共资源等方面优先考虑未成年人的利益和需求，在处理未成年人事务中始终把未成年人权益和全面健康成长放在首位，确保未成年人依法得到特殊、优先保护。
　　--坚持系统谋划统筹推进。加强全局谋划、统筹布局、整体推进，有效发挥各级未成年人保护工作协调机制统筹协调、督促指导作用，着力补短板、强弱项，强化顶层设计、部门协作。坚持未成年人保护工作的政治性、群众性、时代性、协同性，积极推动各方力量参与未成年人保护工作，构建家庭保护、学校保护、社会保护、网络保护、政府保护、司法保护“六位一体”的新时代未成年人保护工作格局。
　　（三）总体目标。
　　到2025年，上下衔接贯通、部门协调联动的未成年人保护工作体制机制基本形成，制度体系逐步健全，与[未成年人保护法](https://www.pkulaw.com/chl/883cecd7fe38636dbdfb.html)相衔接的法律法规体系不断完善，工作力量有效加强，侵害未成年人合法权益案件发生率明显下降，全社会关心关注未成年人健康成长的氛围显著增强。到2035年，与我国经济社会发展相适应、与人口发展战略相匹配的未成年人保护工作体系全面建立，加强未成年人保护工作成为各部门、各行业和社会各界的行动自觉，成为全面建成社会主义现代化国家的显著标志之一，未成年人的生存权、发展权、受保护权、参与权等权利得到更加充分保障。

　　**二、**重点任务
　　（一）强化家庭监护责任。
　　1.加强家庭监护指导帮助。巩固和强化家庭监护主体责任，加大宣传培训和健康教育力度，指导未成年人的父母或者其他监护人依法履行监护职责，抚养、教育和保护未成年人。推动构建家庭教育指导服务体系，加强社区家长学校、家庭教育指导服务站点建设，为未成年人的父母或其他监护人、被委托人每年提供不少于一次公益性家庭教育指导服务。婚姻登记机关办理离婚登记及人民法院审理家事案件时涉及未成年子女的，要对当事人进行未成年人保护相关家庭教育指导。
　　2.完善家庭监护支持政策。全面落实产假等生育类假期制度和哺乳时间相关规定，鼓励有条件的地区探索开展育儿假试点。加强家庭照护支持指导，增强家庭科学育儿能力。有条件的地区，探索对依法收养孤儿和残疾儿童、非生父母履行监护权的家庭在水电气等公共服务方面给予优惠。地方政府在配租公租房时，对符合当地住房保障条件且有未成年子女的家庭，可根据其未成年子女数量在户型选择方面给予适当照顾。推进儿童福利机构拓展集养、治、教、康和专业社会工作服务于一体的社会服务功能，探索向社会残疾儿童提供服务。
　　3.推进家庭监护监督工作。指导村（居）民委员会等相关组织对未成年人的父母或者其他监护人履行监护情况开展监督。村（居）民委员会等相关组织发现未成年人的父母或者其他监护人拒绝或者怠于履行监护责任时，要予以劝阻、制止或者批评教育，督促其履行监护职责；情节严重导致未成年人处于危困状态或造成严重后果的，要及时采取保护措施并向相关部门报告。
　　4.依法处置监护人侵害未成年人权益行为。公安机关接到报告或者公安机关、人民检察院、人民法院在办理案件过程中发现未成年人的父母或者其他监护人存在不依法履行监护职责或者侵犯未成年人合法权益的，应当予以训诫，并可以责令其接受家庭教育指导。对监护人严重损害未成年人身心健康及合法权益，或者不履行监护职责致未成年人处于危困状态等监护侵害行为，依法督促、支持起诉。加强宣传引导和警示教育，及时向社会公布监护人侵害未成年人权益行为处置情况案例。
　　（二）加强学校保护工作。
　　5.加强未成年人思想道德教育。指导学校深入开展共产主义、中国特色社会主义和中国梦学习宣传教育，坚持立德树人，培育和践行社会主义核心价值观，引导广大未成年人听党话、跟党走，养成良好思想品德和行为习惯。指导学校加强新修订的《[中华人民共和国未成年人保护法](https://www.pkulaw.com/chl/883cecd7fe38636dbdfb.html)》等法律法规宣传教育，深入开展未成年人法治教育，提升学生法治意识。深化团教协作，强化少先队实践育人作用，加强未成年人思想道德引领。
　　6.健全学校保护制度。制定《[未成年人学校保护规定](https://www.pkulaw.com/chl/65e0f07394eecfb6bdfb.html)》，整合、完善学校保护制度体系。完善校园安全风险防控体系和依法处理机制，加强校园周边综合治理。提高学生安全意识和自我防护能力，开展反欺凌、交通安全、应急避险自救、防范针对未成年人的犯罪行为等安全教育。积极发展公共交通和专用校车，解决学生上下学乘车难问题，使用校车的学校要加强校车安全管理和使用。强化校园食品安全管理，严格落实校长（园长）集中用餐陪餐、家长代表陪餐、用餐信息公开等制度。严厉打击涉及学校和学生安全的违法犯罪行为。推动落实义务教育学校课后服务全覆盖，与当地正常下班时间相衔接，解决家长接学生困难问题。
　　7.有效防范学生欺凌。进一步完善考评机制，将学生欺凌防治工作纳入责任督学挂牌督导范围、作为教育质量评价和工作考评重要内容。建立健全学生欺凌报告制度，制定学生欺凌防治工作责任清单，压实岗位责任。指导学校定期全面排查，及时发现苗头迹象或隐患点，做好疏导化解工作。完善校规校纪，健全教育惩戒工作机制，依法依规处置欺凌事件。
　　8.创新学校保护工作机制。建立学校保护工作评估制度，评估结果纳入学校管理水平评价和校长考评考核范围。严格落实教职员工准入查询性侵违法犯罪信息制度。充分发挥“法治副校长”、“法治辅导员”作用，常态化开展“法治进校园”、组织模拟法庭、以案释法、开设法治网课等多样化法治教育和法治实践活动，教育引导未成年人遵纪守法。依托中小学校、社区建设少年警校，加强对未成年人的法治教育、安全教育。引入专业力量参与学生管理服务，有条件的地方，可通过建立学校社会工作站、设立社会工作岗位、政府购买服务等方式，推进学校社会工作发展。
　　（三）加大社会保护力度。
　　9.有效落实强制报告制度。指导国家机关、村（居）民委员会、密切接触未成年人的单位、组织及其工作人员有效履行侵害未成年人事件强制报告义务，提升识别、发现和报告意识与能力。建立强制报告线索的受理、调查、处置和反馈制度。加强强制报告法律法规和政策措施的宣传培训和教育引导工作。依法依规对未履行报告义务的组织和个人予以惩处。
　　10.切实发挥群团组织作用。共青团组织要大力推动实施[中长期青年发展规划](https://www.pkulaw.com/chl/c9ba542878e8ad32bdfb.html)，依托“青年之家”、“12355青少年服务台”、“青少年维权岗”等阵地有效维护青少年发展权益。妇联组织要加强对未成年人的父母或其他监护人、被委托人的家庭教育指导，依托“儿童之家”等活动场所，为未成年人保护工作提供支持。残联组织要加强残疾未成年人权益保障，落实残疾儿童康复救助制度，指导有条件的地方，扩大残疾儿童康复救助年龄范围，放宽对救助对象家庭经济条件的限制。工会组织要积极开展职工未成年子女关爱服务，推动用人单位母婴设施建设。关心下一代工作委员会等单位、组织要在职责范围内协助相关部门做好未成年人保护工作。
　　11.积极指导村（居）民委员会履行法定职责。指导村（居）民委员会落实专人专岗负责未成年人保护工作的法定要求，每个村（社区）至少设立一名儿童主任，优先由村（居）民委员会女性委员或村（社区）妇联主席兼任，儿童数量较多的村（社区）要增设补充儿童主任。推进村（社区）少先队组织建设。持续推进“儿童之家”建设。鼓励村（居）民委员会设立下属的未成年人保护委员会。指导村（居）民委员会落实强制报告和家庭监护监督职责，提升发现报告能力。加强村（社区）未成年人活动场所和设施建设，推进村（社区）党群服务中心、文化活动室等服务设施向未成年人开放。指导村（居）民委员会组织开展未成年人保护相关政策宣讲、知识培训活动。
　　12.加强未成年人保护领域社会组织建设。培育和发展未成年人保护领域社会组织，到2025年，实现未成年人保护专业性社会组织县（市、区、旗）全覆盖。大力发展未成年人保护领域专业社会工作和志愿服务，充分发挥社会工作者在未成年人保护工作中资源链接、能力建设、心理干预、权益保护、法律服务、社会调查、社会观护、教育矫治、社区矫正、收养评估等专业优势，积极引导志愿者参与未成年人保护工作。健全未成年人保护领域慈善行为导向机制，依托全国儿童福利信息系统、全国慈善信息公开平台等加强数据共享和供需对接，引导公益慈善组织提供个性化、差异化、有针对性的服务。
　　（四）完善网络保护工作。
　　13.完善未成年人网络保护法规政策体系。加快推动出台未成年人网络保护条例，完善配套政策，净化未成年人网络环境，保障未成年人网络空间安全，保护未成年人合法网络权益，构建网络环境保护长效机制。推动制定未成年人网络保护行业规范和行为准则。加强涉未成年人网课平台和教育移动互联网应用程序规范管理，完善未成年人网课平台备案管理制度。
　　14.加强未成年人个人信息网络保护。指导监督网络运营者有效履行未成年人个人信息网络保护的平台责任，严格依照法律规定和用户协议收集和使用未成年人个人信息。指导网络运营者对未成年人及其监护人提出的更正、删除未成年人网上个人信息的诉求，依法依规予以配合。严厉打击通过网络以文字、图片、音视频等形式对未成年人实施侮辱、诽谤、猥亵或恶意损害形象等网络欺凌行为，指导网络运营者及时配合制止网络欺凌行为并防止信息扩散。
　　15.加强防止未成年人网络沉迷工作。规范网络游戏、网络直播和网络短视频等服务，有效遏制未成年人网络沉迷、过度消费等行为。加强前置审查，严格网络游戏审批管理。严格实行网络游戏用户账号实名注册制度，推动建立统一的未成年人网络游戏电子身份认证系统。有效控制未成年人使用网络游戏时段、时长，规定时间内不得以任何形式为未成年人提供游戏服务。严格规范向未成年人提供付费服务。加强中小学生手机管理，推进未成年学生在校专心学习。
　　（五）强化政府保护职能。
　　16.有效落实政府监护职责。加强政府监护体制机制建设，提高长期监护专业化服务水平，建立健全临时监护工作制度。建立监护评估制度，建立健全由民政部门指定监护人和终止临时监护情形时监护人的监护能力评估工作规范，科学评判其履行监护职责的能力和条件，推动监护评估规范化专业化。完善因突发事件影响造成监护缺失未成年人救助保护制度措施。进一步健全孤儿保障制度，建立基本生活保障标准动态调整机制。
　　17.加强困境未成年人关爱服务。加强困境未成年人分类保障，分类实施困境未成年人保障政策。将符合条件的未成年人纳入最低生活保障、特困人员救助供养等社会救助范围，加强对困难家庭的重病、重残未成年人生活保障工作。合理确定事实无人抚养儿童生活补助标准，对符合条件的事实无人抚养儿童按规定落实医疗救助政策。结合实施乡村振兴战略深化农村留守儿童关爱服务，完善义务教育控辍保学工作机制。进一步落实家庭经济困难儿童教育资助政策和义务教育阶段“两免一补”政策。
　　18.建设高质量教育体系。坚持教育公益性原则，推进基本公共教育服务均等化，推动义务教育优质均衡发展和城乡一体化。保障农业转移人口随迁子女平等享有基本公共教育服务。完善普惠性学前教育和特殊教育、专门教育保障机制，鼓励高中阶段学校多样化发展。办好每所学校，关心每名学生成长，坚决克服唯分数、唯升学倾向。规范校外培训，切实减轻中小学生过重校外培训负担。
　　19.加强未成年人健康综合保障。完善医疗卫生和医疗保障制度，确保未成年人享有基本医疗、卫生保健服务。加强儿童早期发展服务，推动建立医疗机构对儿童视力、听力、肢体、智力残疾和儿童孤独症早期筛查、诊断、干预和政府康复救助衔接机制，深入开展重点地区儿童营养改善等项目。做好未成年人基本医疗保障工作，统筹基本医疗保险、大病保险、医疗救助三重制度，实施综合保障。鼓励有条件的地方研究将基本的治疗性康复辅助器具逐步纳入基本医疗保险支付范围。加强未成年人心理健康教育和服务。重视未成年人早期视力保护，加强综合防控儿童近视工作，及时预防和控制近视的发生与发展。加强中小学生睡眠管理工作，保证中小学生享有充足睡眠时间。切实加强未成年人肥胖防控工作。
　　20.推进婴幼儿照护服务。发展普惠托育服务体系，加大对社区婴幼儿照护服务支持力度。遵循婴幼儿发展规律，完善有关政策法规体系和标准规范，促进婴幼儿照护服务专业化、规范化建设。加强托育机构监督管理，做好卫生保健、备案登记等工作，积极构建综合监管体系。加快培养婴幼儿照护服务专业人才，大力开展职业培训，增强从业人员法治意识。切实强化和落实各方面责任，确保婴幼儿安全和健康。
　　21.加强和创新未成年人成长社会环境治理。构建未成年人成长社会环境治理联合执法机制，加大执法力度。落实未成年人入住旅馆、宾馆、酒店的核查与报告制度。加大对营业性歌舞娱乐场所、酒吧、互联网上网服务营业场所违规接待未成年人行为的处罚力度。落实密切接触未成年人行业违法犯罪信息准入查询制度。严格禁止向未成年人销售烟（含电子烟）、酒、彩票或者兑付彩票奖金。依法依规及时清理中小学校、幼儿园、托育机构周边设置的营业性娱乐场所、酒吧、互联网上网服务营业场所及烟（含电子烟）、酒、彩票销售网点。对部分儿童用品依法实施强制性产品认证管理，保障未成年人健康安全。加大互联网上涉及未成年人的重点应用服务的整治和查处力度，加强监管，督促企业切实落实针对未成年人保护的各项措施。督促中小学校、幼儿园、婴幼儿照护服务机构、线下教育培训机构、游乐园等未成年人集中活动场所落实安全主体责任。推进未成年人文身治理工作。做好未满十六周岁辍学学生劝返复学工作。加大对未成年人违法婚姻的治理力度，防止未成年人早婚早育现象。
　　（六）落实司法保护职责。
　　22.依法妥善办理涉未成年人案件。坚持“教育、感化、挽救”方针和“教育为主、惩罚为辅”原则，严格落实未成年人刑事案件特别程序，依法惩戒和精准帮教相结合，促进未成年人顺利回归社会。办理未成年人遭受性侵害或者暴力伤害案件，施行“一站式取证”保护机制。对于性侵害未成年人犯罪，公安、检察部门积极主动沟通，询问被害人同步录音录像全覆盖。对涉案未成年人实施必要的心理干预、经济救助、法律援助、转学安置等保护措施，积极引导专业社会工作者参与相关保护工作。
　　23.加强少年法庭建设。深化涉未成年人案件综合审判改革，将与未成年人权益保护和犯罪预防关系密切的涉及未成年人的刑事、民事及行政诉讼案件纳入少年法庭收案范围。审理涉及未成年人的案件，从有利于未成年人健康成长的角度出发，推行社会调查、社会观护、心理疏导、司法救助、诉讼教育引导等制度，依法给予未成年人特殊、优先保护。加强未成年人法律援助，积极开展司法救助，及时帮扶司法过程中陷入困境的未成年人，充分体现司法的人文关怀。
　　24.深化未成年人检察法律监督。依法对涉及未成年人的诉讼活动、未成年人重新犯罪预防工作等开展法律监督。及时对未尽到未成年人教育、管理、救助、看护等保护职责的有关单位提出建议。进一步加强涉及未成年人刑事、民事、行政、公益诉讼检察业务统一集中办理工作。开展未成年人刑事案件羁押必要性审查，对涉及未成年人刑事案件立案、侦查和审判活动，以及涉及未成年人民事诉讼、行政诉讼和执行活动进行监督。开展未成年人监管及未成年人社区矫正活动监督。加大对侵犯未成年人合法权益案件督促、支持相关组织和个人代为提起诉讼的力度，涉及公共利益的依法提起公益诉讼。推动未成年人司法保护协作机制和社会支持体系建设。
　　25.严厉打击涉未成年人违法犯罪行为。依法严惩利用未成年人实施黑恶势力犯罪，对拉拢、胁迫未成年人参加有组织犯罪的，从严追诉、从重量刑。加强未成年人毒品预防教育，引导未成年人从小认清毒品危害，自觉抵制毒品。依法严厉惩治引诱、纵容未成年人从事吸贩毒活动的违法犯罪分子。落实《中国反对拐卖人口行动计划（2021-2030年）》，预防和惩治拐卖未成年人犯罪行为。预防和打击使用童工违法行为。依法查处生产、销售用于未成年人的假冒伪劣食品、药品、玩具、用具和相关设施设备违法犯罪行为。

　　**三、**保障措施
　　（一）加强组织领导。强化党委领导、政府负责、民政牵头、部门协同、社会参与的未成年人保护工作格局。推动各地党委和政府将未成年人保护工作纳入国民经济和社会发展规划及工作绩效评价。依法将未成年人保护工作纳入乡镇（街道）、村（社区）职责范围。将未成年人保护工作开展情况作为平安建设考核重要内容，落实落细文明城市、文明村镇、文明单位、文明家庭和文明校园创建中未成年人保护相关要求。制定实施《中国儿童发展纲要（2021-2030年）》。按有关规定组织开展未成年人保护工作表彰奖励，对有突出表现的给予表彰。
　　（二）加大工作保障。加强未成年人服务设施建设，建立和改善适合未成年人的活动场所和设施，支持公益性未成年人活动场所和设施的建设和运行。加强未成年人救助保护机构等场所服务设施设备建设。将未成年人保护工作相关经费纳入本级预算。将未成年人关爱服务纳入政府购买服务指导性目录，通过政府购买服务等方式引导社会工作专业服务机构、公益慈善类社会组织为留守儿童、困境儿童等特殊儿童群体提供专业服务。加强民政部本级和地方各级政府用于社会福利事业的彩票公益金对未成年人保护工作的支持。加强未成年人保护科学研究和人才培养。
　　（三）充实工作力量。充实基层未成年人保护工作力量，实现未成年人保护工作一线有机构负责、有专人办事、有经费保障。指导各地根据需要，通过整合相关编制资源、盘活编制存量、推动机构转型等方式加强未成年人救助保护机构建设，承担好需依法临时监护的未成年人收留、抚养等相关工作。指导乡镇（街道）设立未成年人保护工作站，及时办理未成年人保护相关事务。加强儿童督导员、儿童主任专业化建设，鼓励其考取社会工作职业资格。加强未成年人审判组织建设和审判专业化、队伍职业化建设。各级人民法院、人民检察院根据实际需要明确相应机构或者指定人员负责未成年人审判、检察工作。指导基层公安派出所加强未成年人保护工作，根据实际明确相关人员负责未成年人保护工作。
　　（四）深入宣传引导。深入开展未成年人保护工作和《[中华人民共和国未成年人保护法](https://www.pkulaw.com/chl/883cecd7fe38636dbdfb.html)》等法律法规的宣传教育，中央和地方有关新闻媒体可设置专栏，基层单位要充分利用所属网站、新媒体、宣传栏等平台，开展全方位、多角度、立体式宣传活动，贯彻落实《新时代爱国主义教育实施纲要》、《新时代[公民道德建设实施纲要](https://www.pkulaw.com/chl/9853b5dc10a7e00cbdfb.html)》，营造全社会关心支持未成年人保护工作的良好氛围。进一步规范新闻媒体对涉及未成年人相关热点事件的宣传报道，传播社会正能量。
　　（五）强化监督检查。加强对未成年人保护工作的监督检查，建立健全业务指导、督促检查和重大事项通报制度。各级未成年人保护工作协调机制设立专兼职相结合的未成年人权益督查专员，负责牵头对各地各部门开展未成年人保护工作情况进行督促检查，对存在的突出问题以及侵害未成年人权益的恶性案件、重大事件进行跟踪指导、挂牌督办、限时整改。

© 北大法宝：（[www.pkulaw.com](https://www.pkulaw.com/)) 专业提供法律信息、法学知识和法律软件领域各类解决方案。北大法宝为您提供丰富的参考资料，正式引用法规条文时请与标准文本核对。
欢迎查看所有[产品和服务](https://www.pkulaw.com/helps/2.html)。法宝快讯：[如何快速找到您需要的检索结果？](https://www.pkulaw.com/helps/70.html)[法宝](https://www.pkulaw.com/helps/69.html)V6有何新特色？

扫描二维码阅读原文

**原文链接：**<https://www.pkulaw.com/chl/c0cfb449e587319fbdfb.html>
